# δ-Catenin Is Genetically and Biologically Associated with Cortical Cataract and Future Alzheimer-Related Structural and Functional Brain Changes

**DOI:** 10.1371/journal.pone.0043728

**Published:** 2012-09-11

**Authors:** Gyungah Jun, Juliet A. Moncaster, Carolina Koutras, Sudha Seshadri, Jacqueline Buros, Ann C. McKee, Georges Levesque, Philip A. Wolf, Peter St. George-Hyslop, Lee E. Goldstein, Lindsay A. Farrer

**Affiliations:** 1 Department of Medicine (Biomedical Genetics), Boston University Schools of Medicine and Public Health, Boston, Massachusetts, United States of America; 2 Department of Ophthalmology, Boston University Schools of Medicine and Public Health, Boston, Massachusetts, United States of America; 3 Department of Biostatistics, Boston University Schools of Medicine and Public Health, Boston, Massachusetts, United States of America; 4 Department of Psychiatry, Boston University Schools of Medicine and Public Health, Boston, Massachusetts, United States of America; 5 Department of Neurology, Boston University Schools of Medicine and Public Health, Boston, Massachusetts, United States of America; 6 Department of Pathology & Laboratory Medicine, Boston University Schools of Medicine and Public Health, Boston, Massachusetts, United States of America; 7 Department of Epidemiology, Boston University Schools of Medicine and Public Health, Boston, Massachusetts, United States of America; 8 Framingham Heart Study, Boston University Schools of Medicine and Public Health, Boston, Massachusetts, United States of America; 9 Boston University Alzheimer's Disease Center, Boston University Schools of Medicine and Public Health, Boston, Massachusetts, United States of America; 10 Tanz Centre for Research in Neurodegenerative Diseases, University of Toronto, Toronto, Ontario, Canada; 11 Geriatric Research Education Clinical Center, Bedford Veterans Administration Hospital, Bedford, Massachusetts, United States of America; 12 Neurosciences Research Centre-CHUL, Université Laval, Québec, Canada; 13 Cambridge Institute for Medical Research, University of Cambridge, Cambridge, United Kingdom; University of Kentucky, United States of America

## Abstract

Multiple lines of evidence suggest that specific subtypes of age-related cataract (ARC) and Alzheimer disease (AD) are related etiologically. To identify shared genetic factors for ARC and AD, we estimated co-heritability of quantitative measures of cataract subtypes with AD-related brain MRI traits among 1,249 members of the Framingham Eye Study who had a brain MRI scan approximately ten years after the eye exam. Cortical cataract (CC) was found to be co-heritable with future development of AD and with several MRI traits, especially temporal horn volume (THV, ρ = 0.24, P<10^−4^). A genome-wide association study using 187,657 single nucleotide polymorphisms (SNPs) for the bivariate outcome of CC and THV identified genome-wide significant association with *CTNND2* SNPs rs17183619, rs13155993 and rs13170756 (P<2.6×10^−7^). These SNPs were also significantly associated with bivariate outcomes of CC and scores on several highly heritable neuropsychological tests (5.7×10^−9^≤P<3.7×10^−6^). Statistical interaction was demonstrated between rs17183619 and *APP* SNP rs2096488 on CC (P = 0.0015) and CC-THV (P = 0.038). A rare *CTNND2* missense mutation (G810R) 249 base pairs from rs17183619 altered δ-catenin localization and increased secreted amyloid-β_1–42_ in neuronal cell culture. Immunohistopathological analysis of lens tissue obtained from two autopsy-confirmed AD subjects and two non-AD controls revealed elevated expression of δ-catenin in epithelial and cortical regions of lenses from AD subjects compared to controls. Our findings suggest that genetic variation in delta catenin may underlie both cortical lens opacities in mid-life and subsequent MRI and cognitive changes that presage the development of AD.

## Introduction

Alzheimer disease (AD) and age-related cataract (ARC) are common age-related disorders. Approximately 5.4 million Americans have AD including 13% of people ages 65 and older and nearly 43% of people ages 85 and older [1]. An estimated 20.5 million persons aged 40 years and older in the U.S. show some evidence of ARC [2]. Both AD and ARC are highly heritable [3], [4]. Numerous rare and common genetic variants have been robustly associated with AD [3], [5]–[8], and to a lesser extent with ARC [9], but much of the genetic risk of these disorders is still unexplained.

Numerous lines of evidence suggest common factors linking AD-associated pathology in the brain and lens. Comparing aged controls with AD patients, Goldstein et al. observed amyloid-β (Aβ) deposits exclusively in AD lenses in the cytoplasm of deep cortical lens fiber cells [10]. A subsequent study demonstrated increased deposition of Aβ in lens and distinctive deep cortical localization in persons with Down Syndrome, a common chromosomal disorder that is invariantly associated with early-onset age-dependent AD neuropathology resulting from APP gene triplication and Aβ overexpression [11]. Supranuclear and deep cortical cataract has been documented in transgenic mice expressing human Aβ [12], [13], and fiber cell membrane defects similar to those described in human cataracts have been observed in transgenic mice carrying a complete copy of human APP from the Down Syndrome critical region of chromosome 21 [14]. In addition, AD-linked Aβ accumulation and light-scattering cytosolic Aβ microaggregate formation co-localize with amyloid pathology and subequatorial supranuclear and deep cortical fibers of human subjects with late-onset AD and Down syndrome associated AD [15], [16].

Genetic association studies support common pathways linking Alzheimer disease (AD) and specific cataract subtypes. Variants in the kinesin light chain 1 gene (*KLC1)* are associated with AD and age-related cataract [17]–[19]. A mutation in the gene encoding ephrin receptor A2 (*EPHA2*) correlates with age-dependent progression of cortical cataract [20], and SNPs in *EPHA1* are significantly associated with AD [6], [21]. In addition, the gene for one of ligands of *EPHA1* and *EPHA2*, *EFNA5*, is expressed in multiple brain regions and associated with hippocampal atrophy [22], enhances recruitment of β-catenin to N-cadherin when bound to the *EPHA2* receptor [20], and causes cataract when knocked out in mice [23].

We hypothesized that common genetic mechanisms govern age-related changes in lens and brain, and that these changes can be detected earlier in the lens. In the current study, we demonstrated that measures of cortical lens opacity in midlife and neurodegeneration in later life are heritable and correlated among participants in the Framingham Study, a population-based, family-structured cohort. To test our hypothesis, we performed a GWAS of these traits in the same sample using a method which considered eye and brain measures as a bivariate outcome. The role of a gene, *CTNND2*, which emerged from the GWAS as genome-wide significant, was then evaluated by immunohistochemistry and expression studies.

## Methods

### Subjects

The Framingham Study is a multi-generation community based study that began as a study of cardiovascular disease in 1948 among 5209 individuals comprising the Original cohort [24]. The Framingham Offspring Study includes 5216 spouses and offspring of the Original cohort members who have been followed since 1971 [25]. Surviving members of this cohort were examined and classified for lens opacities during exams conducted between May 1989 and December 1991 as part of the Framingham Offspring Eye Study (FOES) [26]. Starting in 1999, dementia-free surviving members of both Original and Offspring cohorts were invited to have a magnetic resonance imaging (MRI) examination of the brain. Repeat measurement data were obtained from a second MRI scan performed 2.6 years and 6.2 years later on average in the Original and Offspring cohorts, respectively. For the molecular genetic analysis, we focused on 1249 members of the Offspring cohort for whom ocular, MRI and GWAS data were publicly available (http://www.ncbi.nlm.nih.gov/gap. Accessed 2012 July 2). Subsets of Framingham Study subjects included in each component are shown in Fig. S1. This study was approved by the Boston University Institutional Review Board.

### Phenotypic Evaluation

Ophthalmic examinations in the FOES were performed by two experienced certified ophthalmologists who evaluated the slit lamp photographs obtained through a dilated pupil using a standardized grading system [27]. Both eyes were examined in all study subjects. Nuclear opacification was classified by comparison to a set of standard photographs (grades 0 to 3) according to a validated ordinal rating system [28]. Cortical opacification was measured using a grid to assess opaque area in one-eighth wedges from the circumference of the lens cortex. An ordinal rating of cortical cataract was assigned based on the total number of one-eighth wedges occupied by cortical cataract. Persons with ≥4 positive wedges were assigned a rating of 4 (Fig. S2). Posterior subcapsular cataract (PSC) was assessed by measuring the vertical width of posterior opacification recorded by slit lamp photography (range: 0 to 7 mm). We selected the worse measurement from the right and left eyes. Thirty-three subjects with missing ophthalmic examination data or with a history of cataract surgery were excluded from the analysis.

Details of the MRI scan protocol and procedures for deriving structural volumes and measures of degeneration have been described previously [29]. In brief, volumetric brain MRI measures were obtained using analysis of digital images from the MRI scans. Participants were imaged on a Siemens 1 or 1.5-T MR machine (Siemens Medical, Erlangen, Germany). Digital images were processed by a central laboratory blinded to demographic and clinical identifiers. Quantified MRI measures were derived for frontal lobar volume (FBV), occipital brain volume (OBV), parietal brain volume (PBV), temporal brain volume (TBV), hippocampal volume (HPV), lateral ventricular volume (LVV), temporal horn volume (THV), and white matter hyperintensity volume (WMHV). HPV information was unavailable for 43 (2%) of the subjects at the time of the study.

Subjects were administered a neuropsychological test battery using standard protocols and trained examiners. Details of the tests administered and normative values for the Framingham Original and Offspring cohorts have been published [30], [31]. Cognitive test data were selected from the exam at the time the baseline MRI scan was performed.

### Co-heritability Estimation

Heritability (h^2^) in the narrow sense for a quantitative trait is the ratio of variance contributed by the additive genetic effects to the total phenotypic variance and can be estimated from family data by analysis of intraclass correlations (r). Heritability based on analysis of data obtained among siblings is calculated using the formula: h^2^ = 2r. Co-heritability is the ratio of analogous covariance components between two traits [32]. It can be estimated from analysis of cross-trait sibling correlations. A cross-trait sibling correlation is the average of the correlation of the first trait in sibling A with the second trait in sibling B and the correlation of the second trait in sibling A with the first trait in sibling B. By contrast, self correlation is the correlation between two traits within individuals. The design for estimating self and cross-trait sibling correlations is illustrated in Fig. S3.

Self and cross-trait sibling correlations of volumetric brain MRI and cataract phenotypes with AD were estimated using the FCOR program in the Statistical Analysis for Genetic Epidemiology (S.A.G.E) software (version 6.0) [33]. FCOR calculates correlations for dichotomous and quantitative traits in extended families using the Pearson product-moment estimator with generalized weights. In addition, FCOR estimates multivariate familial correlations with their asymptotic standard errors without assuming multivariate normality of the traits by using a second-order Taylor series expansion and replacing all correlation parameters with their respective estimates [33], [34].

Raw measurements showing significant self and cross-trait correlations were further analyzed by adjusting for sex and age at exam. Age at blood draw (for DNA extraction) was substituted for age at eye exam because this information was not available for a large proportion of subjects at the time the data were analyzed. This was deemed reasonable since the age- and sex-adjusted residuals for age at blood draw and age at eye exam were highly correlated among individuals who had information for both variables (r = 0.96, P<10^−4^) and the blood draw and eye exam occurred during the same Framingham Study exam cycle. Correlations were also computed for the annual rate of change in the selected MRI traits which was calculated as the difference in the measures between the baseline and second MRI evaluations divided by the number of years between examinations. Because the distributions of many traits were multi-modal, age and sex-adjusted residuals for each trait were derived and normalized, forcing the marginal distribution of the trait to be approximately normally distributed under the null hypothesis, by taking the inverse standard normal transformation of the empirical quantile that was obtained using the formula [*r(y)-1/3*] divided by *(n+1/3)*, where *r(y)* is the rank of residuals, *y* [20]. This approach has been shown to be valid in family-based association tests even with rare variants [20]. Normalized residuals were used for subsequent analyses. The significance threshold accounting for 38 independent tests was set at P = 0.0013.

### Genotyping and Data Cleaning

All available participants were genotyped at Affymetrix (Santa Clara, CA) using the Affymetrix GeneChip® Human Mapping 500K Array Set and 50K Human Gene Focused Panel®. Phenotype and genome-wide association study (GWAS) data including raw genotyping calls and genotypes imputed using the HapMap 2 and 3 reference populations were obtained from the dbGaP website (http://www.ncbi.nlm.nih.gov/gap. Accessed 2012 July 2).

Prior to analysis, SNPs with a call rate less than 98%, SNPs with a minor allele frequency (MAF) less than 2%, and SNPs not in Hardy-Weinberg equilibrium (P<10^−6^) were excluded. Individuals with SNP call rates below 98% among the remaining SNPs, or whose gender as determined by analysis of X-chromosome data using PLINK [35] was inconsistent with the self-reported gender were also excluded. The 354,310 SNPs remaining among 7,966 subjects were then pruned to remove one from each pair of SNPs in high linkage disequilibrium (r^2^>0.8) using PLINK with a window size of 1500 SNPs and a step size of 150. After applying these criteria, 187,657 genotyped autosomal SNPs remained. Familial relationships were verified by examining segregation of marker genotypes within families using the MARKERINFO program in S.A.G.E. After confirming these relationships, marker genotypes showing Mendelian inconsistency were set to missing.

Population substructure was examined among those with self-reported ethnicity of white or Caucasian, first among the complete sample and again within a set of unrelated individuals consisting of one randomly selected individual from each extended pedigree. The complete sample of individuals was used for investigation of population structure using the pruned and cleaned genotypes from the Affymetrix 500K array and the CEU HapMap phase 2 reference data. SNP loadings were prepared using the smartpca script implemented in the EIGENSTRAT package [36]. This initial run was performed to confirm the presence of a single ethnic-specific cluster delineated based on self-declared ethnicity. For evaluation of within-cluster substructure, a set of SNP loadings were prepared using the pruned and cleaned genotypes from the unrelated sample of individuals with the same smartpca script implemented in the EIGENSTRAT package. The SNP weights for each eigenvector from the unrelated sample were then applied to all remaining family members to compute principal components for all individuals in the sample. We did not find any significant association between principal components and any cataract or MRI phenotypes in univariate analyses.

### GWAS Analysis Methods

The SNP pruning strategy described above was employed to reduce computational burden associated with bivariate analysis in extended families. SNPs were coded under an additive model as 0, 1, or 2 with respect to the number of the reference alleles. The coded SNP values were included as covariates in regression models along with variables for polygenic effects and random effects to account for the various constellations of relative pairs. Association of each SNP with a bivariate outcome comprising age and sex-adjusted normalized residuals of quantitative measures for one cataract trait (*ycc*) and one MRI trait (*ymri*) was evaluated using the bivariate extension of the two-level Haseman-Elston (tHE) regression method developed for general pedigree data implemented in the RELPAL program in S.A.G.E [37], [38]. The regression model is denoted as: *ycc_ik_,ymri_ik_ ≃ βx_ik_+bz_ik_+e_ik_* for individual *i* in pedigree *k*; *x, z* and *e* represent the SNP, polygenic and random effects, respectively; and *β* and *b* are coefficients. This approach uses an iterative generalized squares algorithm which can accommodate various family structures and incorporate both individual-level and pedigree-level covariates. We demonstrated previously by simulation that this regression model has high power (>99%) for a SNP with a large effect size (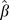
>1.3), even with very rare variant (MAF<0.001), and produces few false positives in large extended families [39]. Some of the pedigrees in the Framingham Study have similar pedigree structures to those included in the simulations.

Nominal P values for association were determined using first-level Wald tests computed by RELPAL. The most significant results (i.e., SNPs with P<10^−5^) in the genome-wide scan were investigated further using data for all known SNPs in the implicated genes including those that were genotyped but had been dropped due to pruning. Additional SNPs in the HapMap 2 and 3 reference panels were imputed using MaCH [40]. All SNPs within 100 kb of top-ranked SNPs not located within genes were analyzed. A quantitative estimate between 0 and 2 representing the dose of the minor allele was used in the analysis instead of imputed genotypes to incorporate the uncertainty of the imputation estimates. We excluded imputed SNPs with low MAF (<2%), not in Hardy-Weinberg equilibrium (P<10^−6^) and low imputation quality (RSQ<0.8). A total of 186,192 genotyped SNPs and 1,465 imputed SNPs for a selected region were analyzed for association. Based on this number of SNPs, the threshold for genome wide significance was determined to be 2.66×10^−7^.

The possibility of a functional role of genes identified by bivariate GWAS in lens opacity and neurodegeneration was evaluated statistically by testing models including the genome wide significant SNP, a SNP from *APP* or *PSEN1*, and an interaction term. The two most significant *APP* genotyped and uncorrelated SNPs (pairwise LD<0.5), each with MAF>0.1, from the bivariate GWAS were tested for interaction with the top *CTNND2* SNP rs17183619. No *PSEN1* SNPs met these criteria and hence were not tested for interaction. The proportion of genetic variance in the bivariate trait explained by rs17183619 was estimated using methods proposed by So and colleagues [41], [42].

### Functional Analysis of the *CTNND2* G810R Mutation

#### Cell Culture

Human Embryonic Kidney 293 cells stably expressing APP Swedish mutation (K595N/M596L of APP695, HEK293-APPsw) were cultured in Dulbecco's modified Eagle's medium (DMEM) supplemented with 10% fetal bovine serum (FBS), 500 µg/ml Geneticin (G418, Invitrogen) and maintained at 37°C in a humidified atmosphere (5% CO2/95% air).

#### Transfection, plasmids and cloning

HEK293-APPsw cells were transfected using Lipofectamine™ 2000 reagent (Invitrogen) according to the manufacturer's protocol. Briefly, plasmid DNA was pre mixed with Lipofectamine (2 µg: 5 µL ratio) in reduced serum media (Opti-MEM®, Invitrogen). DNA-Lipofectamine complexes were added to 80% confluent cells in growth medium without antibiotics for five hours. After incubation, medium was replaced with fresh medium. All assays were performed 24 hours after transfection. pCDNA3 empty vector and pCDNA3His encoding a wild-type human *CTNND2* cDNA were from our plasmid library. Mutant *CTNND2* (glycine to arginine substitution at amino acid position 810) was PCR engineered using pCDNA3His/*CTNND2* as a template and primer sets designed to introduce a missense mutation at codon 810.

#### Western blot

Cell monolayers were lysed in STEN buffer (50 mM Tris [pH 7.6], 150 mM NaCl, 2 mM EDTA, 0.2% NP-40 and 0.5% Triton) supplemented with Complete® protease inhibitor cocktail (Roche). Lysates were pre-cleared of insoluble material by centrifugation and total protein was quantified using BCA reagent (Pierce). Proteins were boiled at 95°C for 5 minutes in Laemmli buffer, separated by molecular weight in 4–12% NuPAGE® Bis-Tris gels (Invitrogen) and transferred to nitrocellulose membranes (Millipore). The membranes were probed for 1 hour with rabbit anti-*CTNND2* (Abcam; 1∶5,000) or mouse anti-tubulin (Santa Cruz; 1∶2,000), washed in Tris Buffered Saline with Tween and re-probed with donkey anti-mouse or donkey anti-rabbit antibodies conjugated to horseradish peroxidase (Santa Cruz; 10,000) for one hour. The proteins were visualized using an ECL reagent (GE).

#### ELISA

The levels of secreted amyloid β (Aβ) peptide were determined by using ELISA Aβ40 and Aβ42 kits (Invitrogen). Briefly, the culture medium was collected with serine protease inhibitor AEBSF (Roche) and immediately assayed or kept at −20°C until tested. Incubation time with Aβ detection antibody was 3 hours. All other steps were performed as described in the manufacturer's protocol. Aβ levels were normalized to total protein and then normalized to the pCDNA3 vector control. Three independent experiments were performed for each condition tested (empty vector, delta-catenin, G810R). Differences in Aβ levels between conditions were evaluated using a two-tailed Student's t test.

#### Microscopy

HEK293-APPsw cells grown on glass coverslips were fixed in 2% paraformaldehyde for 15 minutes, permeabilized in 0.1% saponin for 20 minutes and incubated with rabbit anti-NPRAP antibody (Abcam; 1∶1,000 in 2% BSA and 0.1% saponin in PBS) for 1 hour. Cells were then incubated for another hour with Alexa Fluor 488-conjugated goat anti-rabbit antibody (Invitrogen; 1∶250 in 0.1% saponin in PBS) followed by 15 minutes with DAPI (Sigma; 100 ng/ml). Cells were washed twice in PBS between every step. Coverslips were mounted on slides using Vectashield® mounting medium (Vectorlabs) and observed using an epifluorescence microscope. Semi quantitative analysis of protrusions was performed manually. Images were captured with the 40× objective based on green fluorescence immunoreactivity. Spine-like, filopodia-like and other protrusions on apical segments (branches) of HEK293-APPsw cells were identified based on standard morphological criteria and quantified in areas of equivalent size and magnification. All experiments were performed blinded to the identity of transfected constructs and repeated three times per condition tested (empty vector, δ-catenin or G810R). P values were obtained by two-tailed Student's t test.

### Immunohistochemical Analysis

Eyes from a 68 year-old male and 71 year-old female with neuropathologically-confirmed AD and from two normal male controls ages 68 and 70 were procured from the Boston University Alzheimer Disease Center and National Disease Research Interchange (Philadelphia, PA). Eyes were fixed in 4% paraformaldehyde, embedded in paraffin, and sectioned at 8 um. Sections underwent antigen retrieval using citrate buffer pH 6.0 diluted to the manufacturer's instructions and heated to 90°C for 20 minutes. Tissue sections were immunostained with δ-catenin antibody (SC-81793, Santa Cruz Biotechnology, Inc., Santa Cruz, CA) at 1∶100 dilution and processed by conventional immunohistochemistry (LabVision Autostainer 360, Freemont, CA). Negative controls were run for each antibody. Tissue sections were visualized by brightfield photomicroscopy.

## Results

### Cortical Cataract is Predictive of Future Abnormal Brain Changes

To identify quantitative measures of brain damage that are the best surrogate markers for presymptomatic AD and associated with lens opacity, we examined correlations among these traits within the same individual and between siblings (Table S1, Fig. S3). Although most of the MRI traits were significantly correlated with AD among all Framingham Study subjects who had at least one MRI exam, only TBV, LVV, and THV were significantly co-heritable with future AD after correction for multiple testing (Table S2). Lens opacity information was available for 1249 individuals who participated in the Framingham Eye Offspring Study (FEOS), had at least one brain MRI exam and were on average 51.3 years old at the time of the ophthalmic examination (Table S1, Fig. S1). At the time of the first MRI exam, none of these subjects were demented and only nine (0.7%) had a previous stroke. The mean interval between the eye exam and the first MRI exam was 9.3 years. Our analysis also revealed a significant sib-sib correlation of AD with cortical cataract (CC, 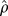 = 0.12, P<10^−4^) and posterior subcapsular cataract (PSC, 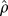 = 0.11, P<10^−4^), but not with nuclear cataract (NC) (Table S2). Heritability and co-heritability estimates were much greater for CC and several MRI traits, particularly THV and LVV, after adjustment for age and sex, and variable normalization (Table 1, Table S3). Taken together, these results suggest that CC and several AD-related brain changes have shared genetic liability or are associated with another variable under genetic influence, and that development of CC may foreshadow development of AD-related brain changes in later life.

**Table 1:** Correlations of cortical cataract with temporal horn volume and lateral ventricular volume.

MRI Trait*	Cross-Trait Correlations with Cortical Cataract†					
	Within an Individual			Between Siblings‡		
	Subjects	COR	P	Sibpairs	COR	P
Temporal Horn Volume (THV)	1249	0.318	<10^−4^	668	0.239	<10^−4^
Lateral Ventricular Volume (LVV)	1249	0.127	1×10^−4^	668	0.153	5×10^−4^
Annual Change of THV	880	−0.128	0.001	497	−0.071	0.116
Annual Change of LVV	880	−0.148	2×10^−4^	497	−0.095	0.043

* Trait values transformed to normalized residuals.

† Adjusted for age and sex.

‡ Calculated by averaging the correlations of cortical cataract in sib 1 with MRI trait in sib 2, and cortical cataract in sib 2 with MRI trait in sib 1.

### Genome-Wide Association

**Figure 1:**
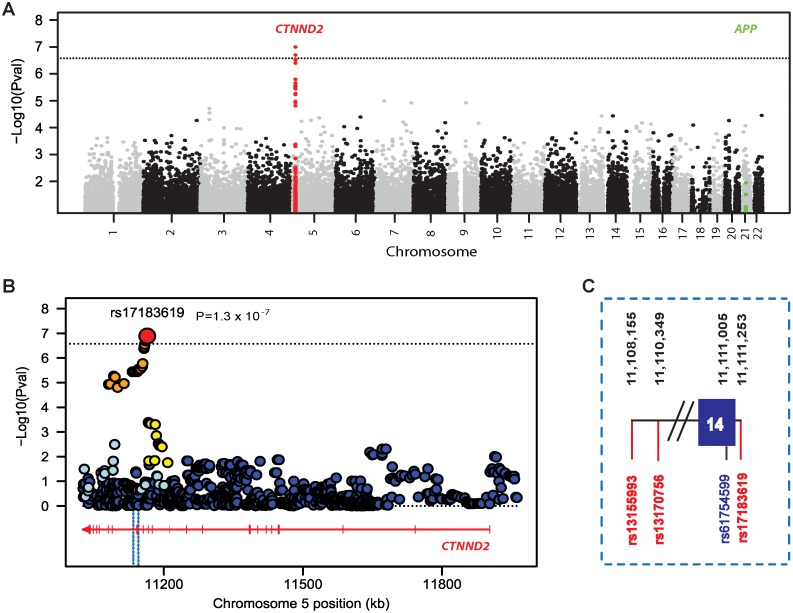
Summary of association results for bivariate analysis of cortical cataract (CC) and temporal horn volume (THV). P-values are expressed as –log_10_(P) (y-axis) for every tested SNP ordered by chromosomal location (x-axis). Genome-wide significance level is shown as a dotted line at P = 2.6×10^−7^. Genomic position was determined using the NCBI database (Build 37.1). (**A**) Manhattan plot showing results for the entire genome. Genotyped and imputed *CTNND2* SNPs are indicated with red dots. (**B**) Regional association plot for the *CTNND2* region on chromosome 5 including the top SNP rs17183619 shown by a red circle. Computed estimates of linkage disequilibrium (r^2^) of SNPs in this region with rs17183619 are shown as orange circles for r^2^≥0.8, yellow circles for 0.5≤r^2^<0.8, light blue circles for 0.2≤r^2^<0.5, and blue circles for r^2^<0.2. The gene structure and reading frame are shown with the red arrow. Exons are denoted with vertical bars on the arrow. The region demarcated by a light blue dotted box includes the most significant SNPs flanking exon 14. (**C**) Expanded view of the region in the light blue box. The three genome-wide significant SNPs indicated in red encompass a non-synonymous variant, rs61754599, indicated in blue that is an amino acid change from glycine to arginine at residue 810. Genomic positions of these SNPs are shown at the top of the panel.

Since CC was much more strongly co-heritable with THV than LVV (Table 1), a genome-wide search was conducted for association of common variants with the bivariate outcome of CC and THV. For this analysis, we used the set of 186,192 genotyped SNPs that remained after applying quality control procedures and a pairwise linkage disequilibrium (LD) cutoff of 0.8 (see Methods). The quantile-quantile (QQ) plot showed that the distribution of P values for the majority of SNPs (i.e., P>10^−5^) met expectation under the null hypothesis (genomic inflation factor lambda = 1.036), whereas more SNPs than expected under the null had P values<10^−5^ suggesting several true associations within this set of SNPs (Fig. S4). While none of the genotyped SNPs were genome-wide significant (threshold P = 2.7×10^−7^), suggestive association (P<5×10^−6^) was observed for CC-THV with three SNPs in *CTNND2* (Table S4). This region was further examined by testing association of CC-THV with 1,465 accurately imputed SNPs (average correlation between actual and imputed genotypes RSQ = 0.9). Genome-wide significance was attained with *CTNND2* SNPs rs17183619, rs13155993, and rs13170756 (Fig. 1A). These SNPs are in high LD (pairwise r^2^>0.9) and the results are supported by strong associations (P<10^−5^) with 19 additional *CTNND2* SNPs (Fig. 1B, Table S5). The association of rs17183619 with CC-THV (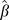
_CC_ = −0.41, 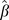
_THV_ = −0.18, P = 1.3×10^−7^) was stronger than the association with either CC (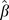 = −0.24, P = 1.1×10^−4^) or THV (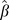 = −0.14, P = 0.017) considered as separate outcomes. The effect direction (indicated by β value sign) was consistent in univariate and bivariate models indicating that the minor allele (G) of rs17183619 is associated with a decrease in both CC and THV. These results suggest that a variant in *CTNND2* influences a process that is more precisely correlated with by degeneration in both the lens and brain than in either tissue considered separately. The proportion of the total co-heritability of CC and THV explained by rs17183619 is 19%.

### *CTNND2* Interacts With APP

Analysis of a model including amyloid precursor protein (*APP*) SNP rs2096488, *CTNND2* SNP rs17183619, and a term for their interaction revealed evidence of a synergistic effect on the bivariate outcome of CC-THV (interaction P = 0.038) (Table 2). There was also significant interaction in the univariate model for CC (P = 0.0015), but not for THV.

**Table 2:** Interaction of *CTNND2* SNP rs17183619 and *APP* SNP rs2096488 in univariate and bivariate models of cortical cataract (CC) and temporal horn volume (THV).

SNP (Reference Allele)	Cortical Cataract		Temporal Horn Volume		Bivariate		
	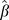	P	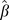	P	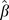 _CC_	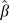 _THV_	P
rs17183619 (G)	−0.266	4.0×10^−5^	−0.123	0.0593	−0.411	−0.193	1.8×10^−7^
rs2096488 (A)	0.046	0.33	0.113	0.0176	0.049	0.093	0.19
Interaction	−0.372	0.0015	0.007	0.95	−0.227	0.18	0.038

Effect estimates (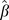) are based on the dosage of the rs17183619 minor allele (G) which has a frequency of 0.153.

### Association of *CTNND2* With Neuropsychological Test Performance

To further explore the influence of variation in *CTNND2* on processes occurring in the eye and brain, the top three SNPs from the GWAS of CC-THV were tested for association with bivariate measures of cortical cataract and performance on the Boston Naming Test (BNT), Immediate Recall (LMI) and Delayed Recall (LMD) portions of Logical Memory subtest of the Wechsler Memory Scale, Hooper Visual Organization Test (HVOT), Wide Range Achievement Tests (WRAT) or the Trail Making Test–B (TMTB) which are among the most heritable neuropsychological tests administered to FHS participants (h^2^>0.7). SNP associations were genome-wide significant with CC-BNT (P<2.4×10^−7^) and CC-WRAT (P<1.0×10^−8^), nearly genome wide significant with CC-LMI (P<6.0×10^−7^), and highly significant with CC-LMD (P<1.3×10^−6^), CC-HVOT (P<1.9×10^−6^) and CC-TMTB (P<3.7×10^−6^). These SNPs were not associated with any of the cognitive tests considered independently of CC (P>0.02).

### A *CTNND2* Functional Variant Alters Amyloid-β (Aβ) Expression

**Figure 2:**
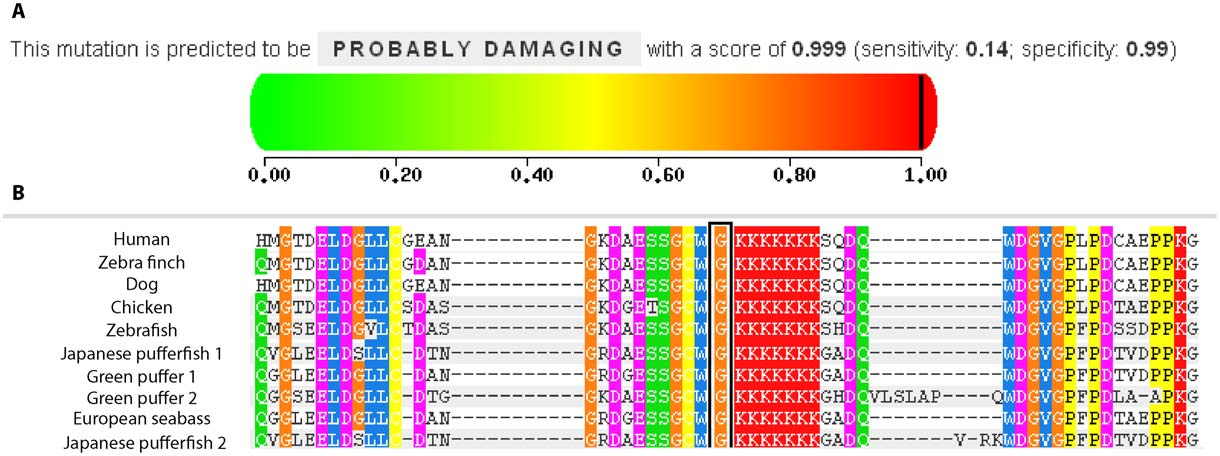
Bioinformatic evaluation of the *CTNND2* non-synonymous coding SNP rs61754599 located 249 base pairs from the top-ranked GWAS result. **A.** Rs61754599 is a missense mutation changing glycine to arginine at residue 810 and was predicted using the PolyPhen2 program (v2.1.0r364) [59] to have a damaging effect on the protein structure. **B.** Comparison of the δ-catenin protein sequence in human, zebra fish, dog, chicken, zebra fish, Japanese puffer fish, green puffer, and European seabass. Columns shaded with the same color denote identity of the same domain across species as determined by phylogenetic clustering of protein sequences. The residue glycine (G) at position 810 (orange column bracketed by the black box) is highly conserved across species.

The top-ranked *CTNND2* SNPs (P<10^−5^) are located in the region encompassing exons 14–16, and the three most significant SNPs flank exon 14 (Fig. 1C). Bioinformatic analysis of this region revealed that the top-ranked SNP, rs17183619, is 249 base pairs upstream of a rare non-synonymous coding SNP (rs61754599) that results in a glycine to arginine missense substitution at residue 810. This missense mutation is predicted to have a deleterious effect on the protein structure (Fig. 2A). The sequence surrounding G810R is highly conserved across several species and bears a signature stretch of basic residues which are required for nuclear function of δ-catenin, the protein encoded by *CTNND2* (Fig. 2B).

**Figure 3:**
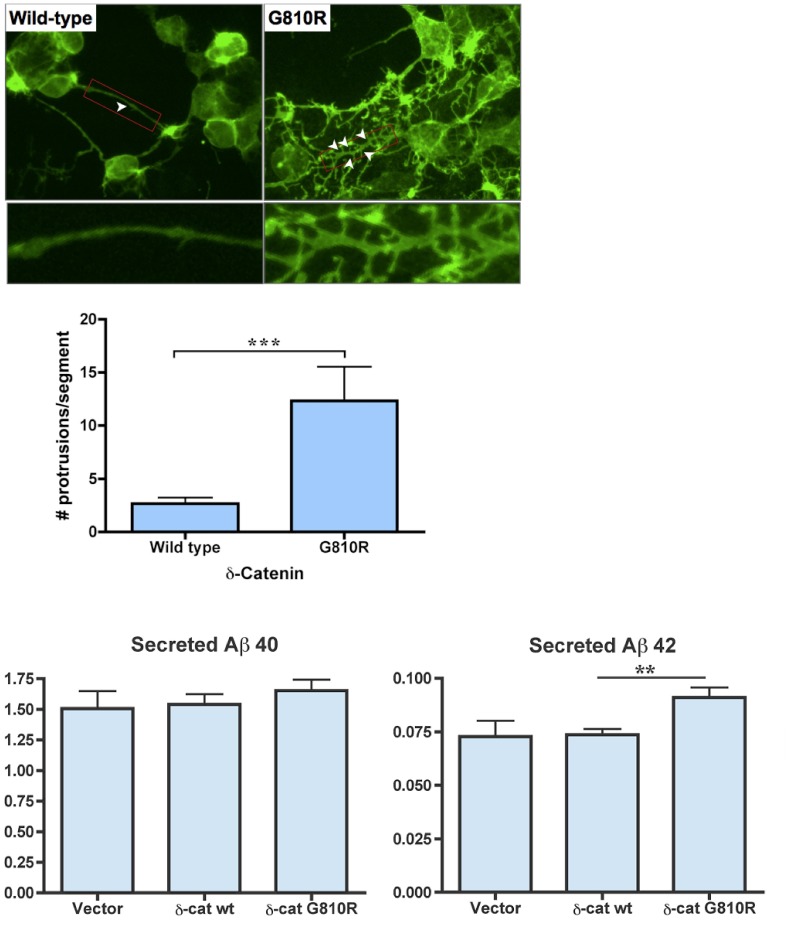
Effect of the *CTNND2* G810R mutation on intracellular distribution of δ-catenin and Aβ secretion in HEK293 cells stably expressing human APP with the Swedish mutation (APP_sw_). **A.** HEK293 APP_sw_ cells were transfected with empty vector, wild type delta-catenin or G810R mutant delta-catenin (n = 3 each). Effect of G810R on cell morphology. In contrast to wild type δ-catenin, mutant δ-catenin is predominantly located at/under the cell surface and G810R mutant cells show a more elaborate network of protrusions (white arrows), suggesting that it may be altering interactions of the plasma membrane with the underlying actin cytoskeleton. Lower panels show detail for apical segments (red rectangles) at a higher magnification. **B.** Semi-quantitative analysis shows significantly more protrusions extending from apical segments in cells with G810R mutation (p = 0.006). Error bars represent the standard deviation. **C.** Effect of G810R on Aβ secretion. Conditioned media was collected at 16 hours and assayed for Aβ_1–40_ and Aβ_1–42_. Aβ concentrations were corrected for total protein levels. Error bars represent the standard deviation. When compared to empty vector controls, wild type δ-catenin had no effect on secreted Aβ levels. In contrast, cells expressing mutant δ-catenin displayed a significant and specific increase in the secretion of Aβ_1–42_ (p = 0.02).

Transfection of the human *CTNND2* cDNA with the *G810R* mutation into HEK293 cells that stably express the human *APP* Swedish mutation (*APP_swe_*) showed that the distribution of mutant δ-catenin is altered predominantly at or near the cell surface (Fig. 3A). Transfected cells expressing the *G810R* mutation had a significantly more elaborate network of protrusions (P = 0.006, Fig. 3B), suggesting that the mutated *CTNND2* gene may alter interactions of the plasma membrane with the underlying actin cytoskeleton. Moreover, transfection with the mutant δ-catenin resulted in a significant and specific increase in Aβ_1–42_ secretion (P = 0.02, Fig. 3C).

### δ-Catenin is Expressed in Human Lens

**Figure 4:**
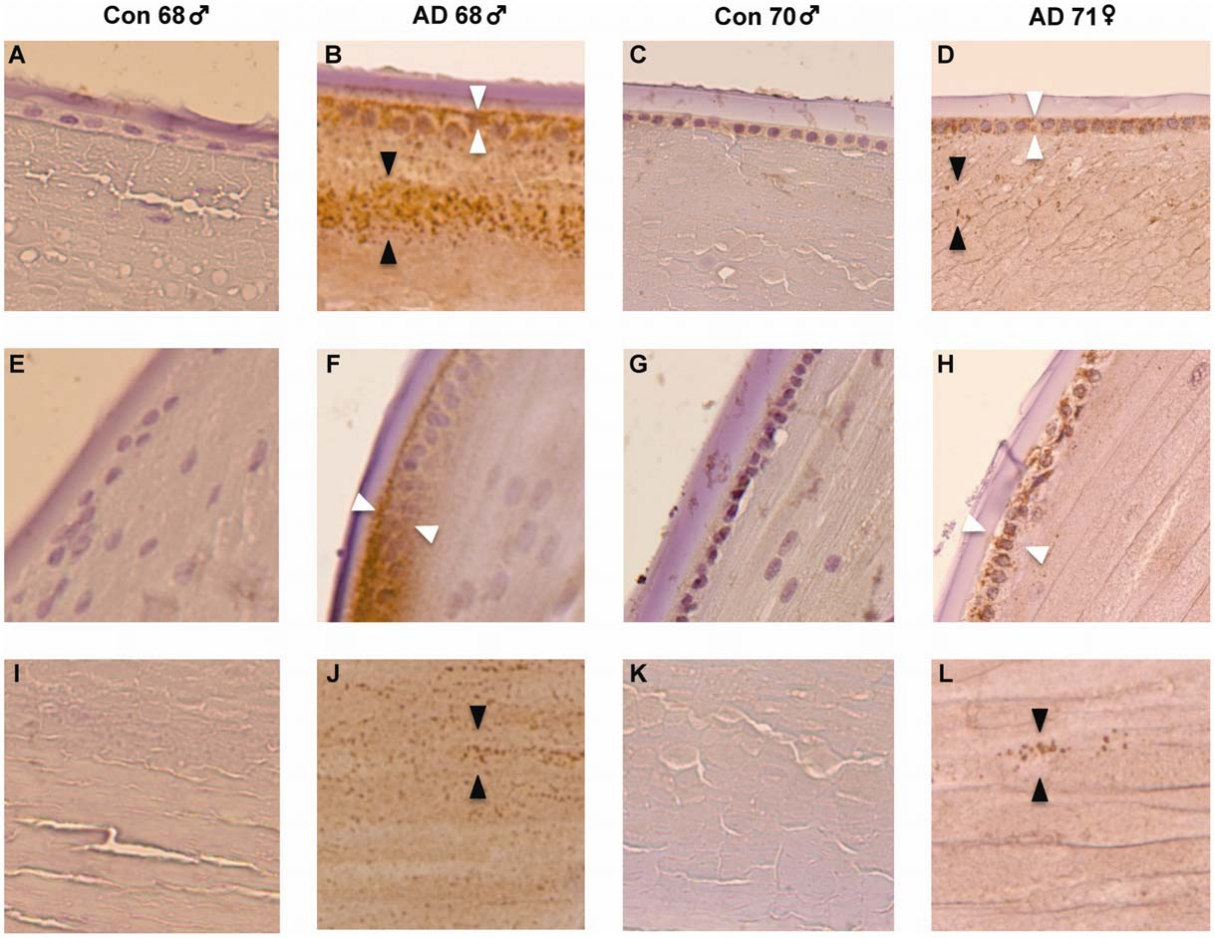
Immunohistochemistry of δ-catenin in the human lens in Alzheimer disease (AD) from a 68 year-old male and 71 year-old female with neuropathologically-confirmed AD and from two normal male controls ages 68 and 70. Staining was observed in the anterior (**Panels A–D**), equatorial (**Panels E–H**) and supranuclear (**Panels I–L**) regions. In the anterior region, intense δ-catenin immunoreactivity was observed in the epithelial cell layer with a striking basolaminar distribution in the AD cases (**Panels B and D**) compared to age-matched controls (**Panels A and C**). Dense punctate δ-catenin immunoreactive deposits were observed in the epithelium (white arrowheads in **Panels B and D**) with laminar preponderance in the subjacent cortex and in the subequatorial and deep cortex of AD lenses (black arrowheads in **Panels B and D**). Dense punctate δ-catenin immunoreactive staining was also observed in the supranuclear region in the AD cases (black arrowheads in **Panels J and L**).

We detected δ-catenin immunoreactivity in the central and bow regions of the lens epithelium and underlying anterior, subequatorial, and deep supranuclear cortical regions of the lens (Fig. 4) and retina (Fig. S5). The tissue localization pattern in postmortem lens from subjects with neuropathologically confirmed AD, but not lens from non-AD controls, was notable for abnormally increased δ-catenin immunoreactivity in the lens epithelium and cortex regions of the lens examined. Moreover, we detected pathological accumulation of δ-catenin immunoreactive product in AD lenses that was detectable as compacted cytosolic deposits with a striking basolaminar preponderance in the anterior and equatorial epithelium and as heterogeneously distributed punctate deposits in the superficial, subequatorial, deep cortical and supranuclear lens subregions. The presence of these pathological δ-catenin immunoreactive accumulations in AD lenses comprises a presumptive biological substrate for locally increased light scattering, lenticular opacification, and frank cataract.

## Discussion

We observed in a sample of Framingham Study participants a correlation of a cortical cataract score measured during adulthood with future development of AD, and with multiple measures of AD-related brain degeneration obtained from both MRI scan and cognitive testing nearly ten years later (Table 1 and Table S2). Our unique GWAS identified genome-wide significant association of three intronic SNPs in *CTNND2* with a bivariate outcome of cortical cataract and volume of the temporal horn, an MRI measurement that strongly correlates with AD progression [43]. This association accounts for 19% of the heritable component of CC-THV. These SNPs were also significantly associated with the bivariate outcomes of cortical cataract and performance on several neuropsychological tests including the BNT, LMI, LMD and TMTB which are strongly associated with future AD risk [44]–[46] evaluated at the same time as the MRI scan, suggesting that variants in *CTNND2* may influence functional as well as structural brain changes. Our analyses also revealed a significant interaction of the top-ranked *CTNND2* SNP (rs17183619) with *APP* SNP rs2096488 on the degree of cortical cataract and on the bivariate outcome of cortical cataract and THV. Interestingly, the interaction between *CTNND2* and *APP* was strongest in the univariate cortical cataract model suggesting the possibility that this interaction may be stronger or is detectable earlier in the lens. In addition, we demonstrated that a rare missense mutation (G810R) located 249 bp from rs17183619 alters the distribution of δ-catenin and Aβ secretion in neuronal cells. Immunohistochemistry experiments using a δ-catenin antibody revealed punctate staining in the cortical and supranuclear region of the lens from autopsy-confirmed AD cases but not from subjects lacking AD-associated neuropathology.

The rarity of the G810R mutation makes it unlikely that this polymorphism explains the association with *CTNND2*, despite its proximity to rs17183619 and demonstrated effect on APP processing. It is more likely that common variants or multiple rare variants in tight linkage disequilibrium with rs17183619 alter the function or expression of *CTNND2* perhaps by affecting splicing or transcription factor binding.

*CTNND2* encodes an adhesive junction-associated protein of the β-catenin superfamily that has been implicated in brain and eye development [47]. The δ-catenin protein is a component of the cadherin-catenin complex that recruits presenilin 1 to cadherins and inhibits Aβ production [48], [49]. SNPs rs17183619 and rs13170756 surround exon 14 that contains the G810R mutation and encodes part of the highly conserved set of 10 armadillo repeat domains in δ-catenin [50]. Armadillo repeat domains 2–10 are necessary and sufficient to mediate the binding of δ-catenin to the hydrophilic loop of presenilin 1 in human brain [51]. Presenilin 1-deficient mice show significantly reduced expression of δ-catenin [52] and mice lacking normal δ-catenin display severe impairments in learning and memory tasks and in synaptic plasticity [53]. Emerging evidence supports a critical role for δ-catenin in dendritic spine maturation and maintenance in the cerebral cortex [54]. Recently, it has been suggested that δ-catenin tethers γ-secretase near synaptic membranes [55], supporting the previously proposed concept that γ-secretase complexes exist at the synapse where they are active and regulate synaptic function [56]. Thus it is possible that variations in *CTNND2* alter synaptic function via a mechanism involving γ-secretase.

The mechanism underling the change in Aβ secretion observed in cells transfected with the G810R mutation warrants further investigation. We have previously shown that δ-catenin interacts with the TM6-TM7 hydrophylic loop domain of presenilin 1 [57], thus providing a plausible mechanism for direct modulation of γ-secretase activity. However, we also observed that the mutant δ-catenin has a markedly different subcellular distribution from the wild type protein in HEK293 cells transfected with the mutant δ-catenin construct, an observation consistent with the striking abnormal basolaminar distribution pattern noted in the epithelial layers of human AD lenses. Taken together, these observations suggest that δ-catenin may be aberrantly concentrated under the plasma membrane rather than in the nucleus in AD lens and brain. It is therefore conceivable that G810R alters intracellular trafficking of membrane proteins such as APP, or alters a signaling/transcriptional pathway that influences γ-secretase activity.

Our study showed only modest association of *CTNND2* with cortical cataract and no evidence for association with measures of brain degeneration when the lens and brain traits were considered independently in univariate analysis. These observations suggest that *CTNND2* accounts for a very small portion of the genetic component of late-onset AD and age-related cortical cataract captured by the phenotype classification system used in the FEOS. Alternatively, AD-linked cortical cataract may be a distinct disorder as suggested by the distinctive subequatorial supranuclear phenotype observed in late-onset AD and Down syndrome [10], [11]. Another explanation is that *CTNND2* variation affects a very specific process or pathway that is best represented by the bivariate measures of degeneration in the lens and brain that are mechanistically related to altered binding of δ-catenin to presenilin 1. This hypothesis is supported by our data showing apparent increased accumulation and abnormal cellular distribution of δ-catenin in lenses from neuropathologically-confirmed AD patients. The significant interaction of *CTNND2* and *APP* SNPs on the univariate measure of cortical cataract highlights the importance of investigating physiological changes in the lens that may precede neuronal loss.

One of the innovative aspects of this study is the application of a bivariate framework to identify genes for co-heritable traits. This approach detected genome-wide significant association of *CTNND2* variants with the bivariate outcomes of cortical cataract and temporal horn volume, whereas association with this gene was not statistically remarkable when these traits were considered separately. Family-based samples are particularly well suited for establishing co-heritability of traits before consideration as a bivariate outcome in genetic association studies. Thus, the Framingham Offspring Study provided a unique opportunity to test our hypotheses because of its family-based design, prospective follow-up of individuals unbiased by selection for ocular or neurological disease, detailed ophthalmological data acquired about ten years prior to the first brain MRI examination, longitudinal monitoring of cognitive status, and availability of GWAS data.

While our results are significant and mechanistically plausible, our study has several limitations. The association findings of *CTNND2* SNPs with quantitative outcomes derived from measures of cortical lens opacification and AD-linked neurodegeneration should be replicated in an independent sample even though they met genome-wide significance criteria. However, to our knowledge there are no other large cohorts having eye measurement data obtained many years before acquisition of brain MRI scan data. For this reason, we validated these findings through genetic association analyses with measures of cognitive performance and by experimental approaches. Our association findings for *CTNND2* with bivariate outcomes including cognitive function are consistent with results from a recent GWAS in a prospectively followed cohort showing near genome-wide significance for association of rate of cognitive decline with rs2973488 [58], a *CTNND2* SNP located 2 kb distal of rs13189742 which was strongly associated with the CC-THV bivariate outcome (Table S5). Another limitation relates to the use of an ordinal variable that precludes inclusion of precataractous lens pathology. Nonetheless, we were able to demonstrate statistically significant association with multiple variables derived from this less precise measure of lens opacity. We also note that meaningful comparisons of co-heritability estimates for clinically ascertained AD with specific cataract and MRI phenotypes in the same sample could not be made as only seven incident AD cases currently exist among the 1249 subjects who participated in both the eye and MRI examination arms of the study. However, analysis of an enlarged sample including 139 AD cases who had the eye exam only revealed significant co-heritability of CC and PSC with AD (Table S2). Thus, the co-heritability estimates for lens and MRI traits are especially remarkable since all of these subjects were dementia-free at the time of the ophthalmic examination and ten years following at the time of the initial MRI examination. Future studies of this cohort are likely to provide additional insight into the relationship of AD pathogenesis in the brain and lens.

Taken together, prior observations and the results from our study support the existence of a pathway leading to AD-linked pathology in the brain and lens, a hypothesis that supports a systemic rather than brain-limited focus for age-dependent AD pathogenesis. This hypothesis is indirectly supported by the epithelial origin of the lens and brain (surface ectoderm and neuroectoderm, respectively) and the long-lived nature of the terminal differentiated cell types affected by AD pathology in the lens and brain. Further investigation is needed to determine the specific molecular and cellular mechanisms underpinning presumptive linkage of AD pathology in these two anatomical compartments. The implication that a genetic variant can alter the function of a protein affecting cortical cataract and AD suggests that these two systemically distinct diseases may be related, thus suggesting possible convergent pathogenic mechanisms. Moreover, δ-catenin, and possibly other members of the cadherin-catenin complex, may provide new therapeutic targets for AD and cortical cataracts. Finally, detection of AD-linked lens pathology could serve as a peripherally accessible biomarker to facilitate discovery, development, evaluation, and implementation of emerging AD therapeutics.

## Supporting Information

Figure S1 Experimental design for computational studies. **A.** Dates and mean ages of Framingham Study Original Cohort and Offspring Cohort participants at the time of the eye and brain MRI exams, and mean age at onset of incident AD cases subsequent to these exams. **B.** Samples included in the co-heritability and bivariate GWAS components of the study. Fewer than 15% of the 5,209 Original Cohort members participated in the eye and MRI exams approximately 43 and 53 years, respectively, after entry into the Framingham Study in 1948. Approximately one-half of the 5,216 Offspring cohort members enrolled in 1971 participated in these exams. Co-heritability analyses included members of both cohorts whereas the GWAS study was limited to Offspring Cohort members since eye, MRI and GWAS data were available for very few individuals from the Original Cohort.
(TIF)

Figure S2 Retroillumination slit lamp photomicrograph of a dilated right and left eyes from a 57-year-old female Framingham Offspring Study participant demonstrating equatorial cortical opacification with cortical spoking and posterior extension.
(TIF)

Figure S3 Heritability and co-heritability estimation. The heritability of a trait is derived from the correlation among siblings, as shown in the diagram by the solid blue and orange arrows. Co-heritability of two traits is derived from the cross-trait sibling correlation which is obtained by averaging the correlation of the first trait in sibling A with the second trait in sibling B and the correlation of the second trait in sibling A with the first trait in sibling B shown by the crossed blue and orange arrows.
(TIF)

Figure S4 Quantile-quantile (Q-Q) plot of observed (y-axis) vs. expected (x-axis) *P*-values from genome-wide association tests for the bivariate outcome of cortical cataract and temporal horn volume. Black dots represent all genotyped SNPs and red dots denote the imputed SNPs from *CTNND2* with P<10^−5^. The number of SNPs (188,629) includes genotyped SNPs (186,192) and imputed SNPs (2,437) from selected gene regions.
(TIF)

Figure S5 δ-catenin in the human retina. **A.** δ-catenin immunostianing in a retina from a 70-year-old female with AD. **B.** same retina in panel **A** stained with hematoxylin and eosin. **C.** δ-catenin immunostianing in a retina from a 50-year-old male control. Abbreviations: VIT, vitreous body; ILM, inner limiting membrane; GCL, granule cell layer; INL, inner nuclear layer; OPL, outer plexiform layer; ONL, outer nuclear layer; PRL, photoreceptor layer; RPE, retinal pigment epithelium; CHO, choroid; SCL, sclera.
(TIF)

Table S1 Sample characteristics.
(DOCX)

Table S2 Trait correlations.
(DOCX)

Table S3 **C**orrelations for cortical cataract with selected MRI traits.
(DOCX)

Table S4 Top-ranked GWAS results (P<10^−5^) with 186,192 genotyped SNPs in bivariate models of cataract and temporal horn volume.
(DOCX)

Table S5 Top-ranked association results (P<10−5) for CTNND2 SNPs with cortical cataract (CC), temporal horn volume (THV), and the bivariate outcome CC-THV.
(DOCX)
