# Supplementary figures and images for: δ-Catenin Is Genetically and Biologically Associated with Cortical Cataract and Future Alzheimer-Related Structural and Functional Brain Changes
Source: PLoS One. 2012 Sep 11;7(9):e43728. doi: 10.1371/journal.pone.0043728 (PMC3439481; doi:10.1371/journal.pone.0043728)

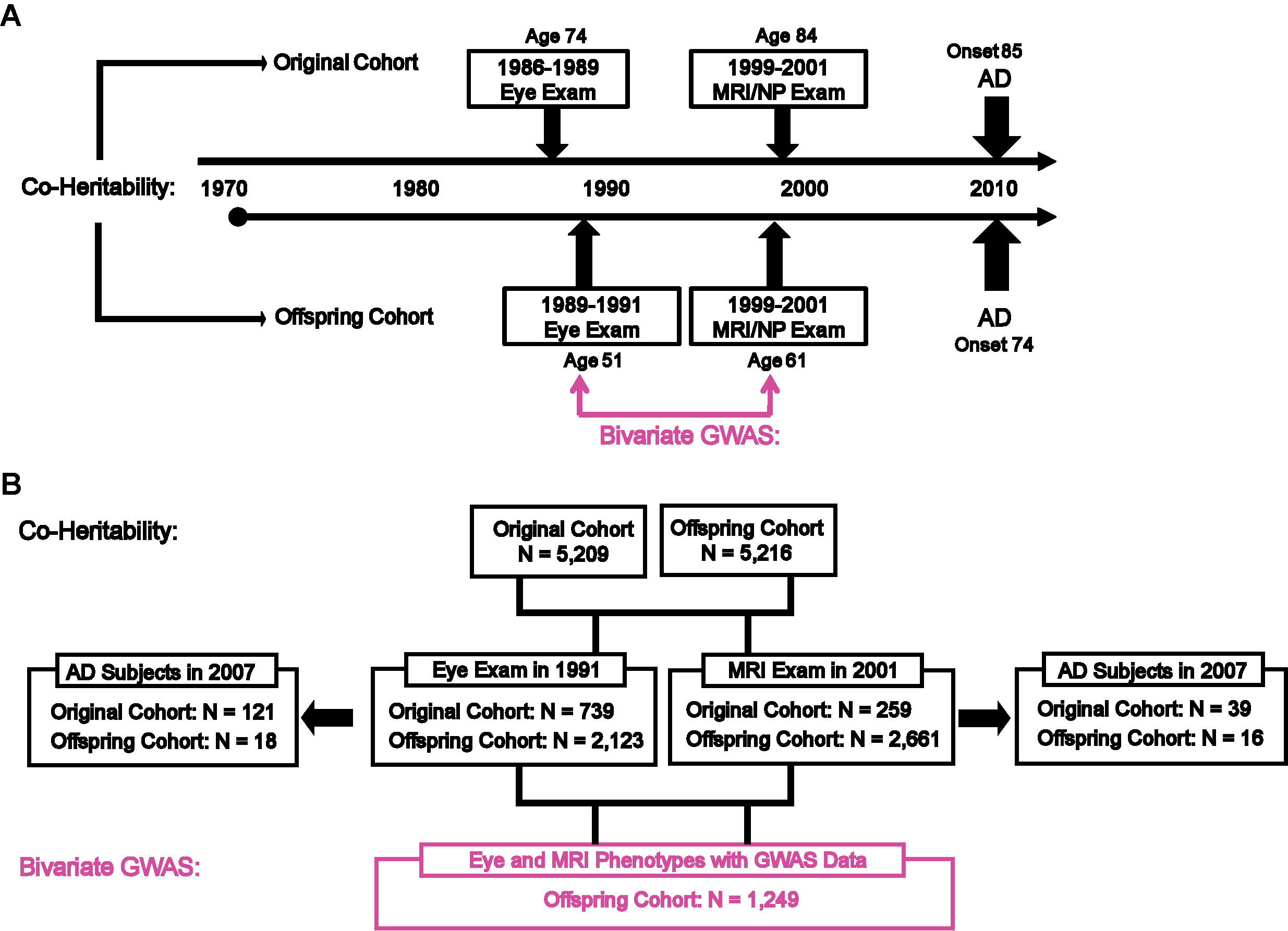

Supplement: Figure S1 — Experimental design for computational studies. A. Dates and mean ages of Framingham Study Original Cohort and Offspring Cohort participants at the time of the eye and brain MRI exams, and mean age at onset of incident AD cases subsequent to these exams. B. Samples included in the co-heritability and bivariate GWAS components of the study. Fewer than 15% of the 5,209 Original Cohort members participated in the eye and MRI exams approximately 43 and 53 years, respectively, after entry into the Framingham Study in 1948. Approximately one-half of the 5,216 Offspring cohort members enrolled in 1971 participated in these exams. Co-heritability analyses included members of both cohorts whereas the GWAS study was limited to Offspring Cohort members since eye, MRI and GWAS data were available for very few individuals from the Original Cohort. (TIF) [file pone.0043728.s001.tif]

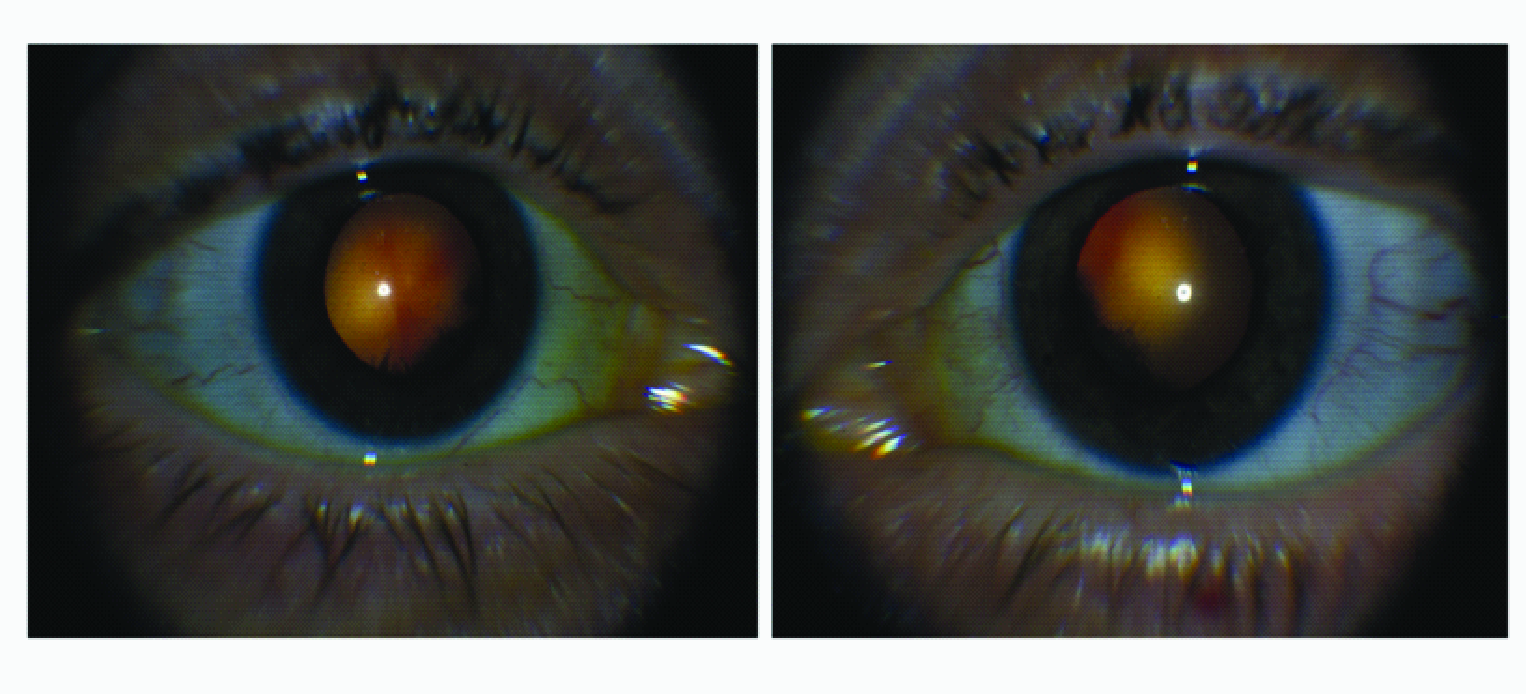

Supplement: Figure S2 — Retroillumination slit lamp photomicrograph of a dilated right and left eyes from a 57-year-old female Framingham Offspring Study participant demonstrating equatorial cortical opacification with cortical spoking and posterior extension. (TIF) [file pone.0043728.s002.tif]

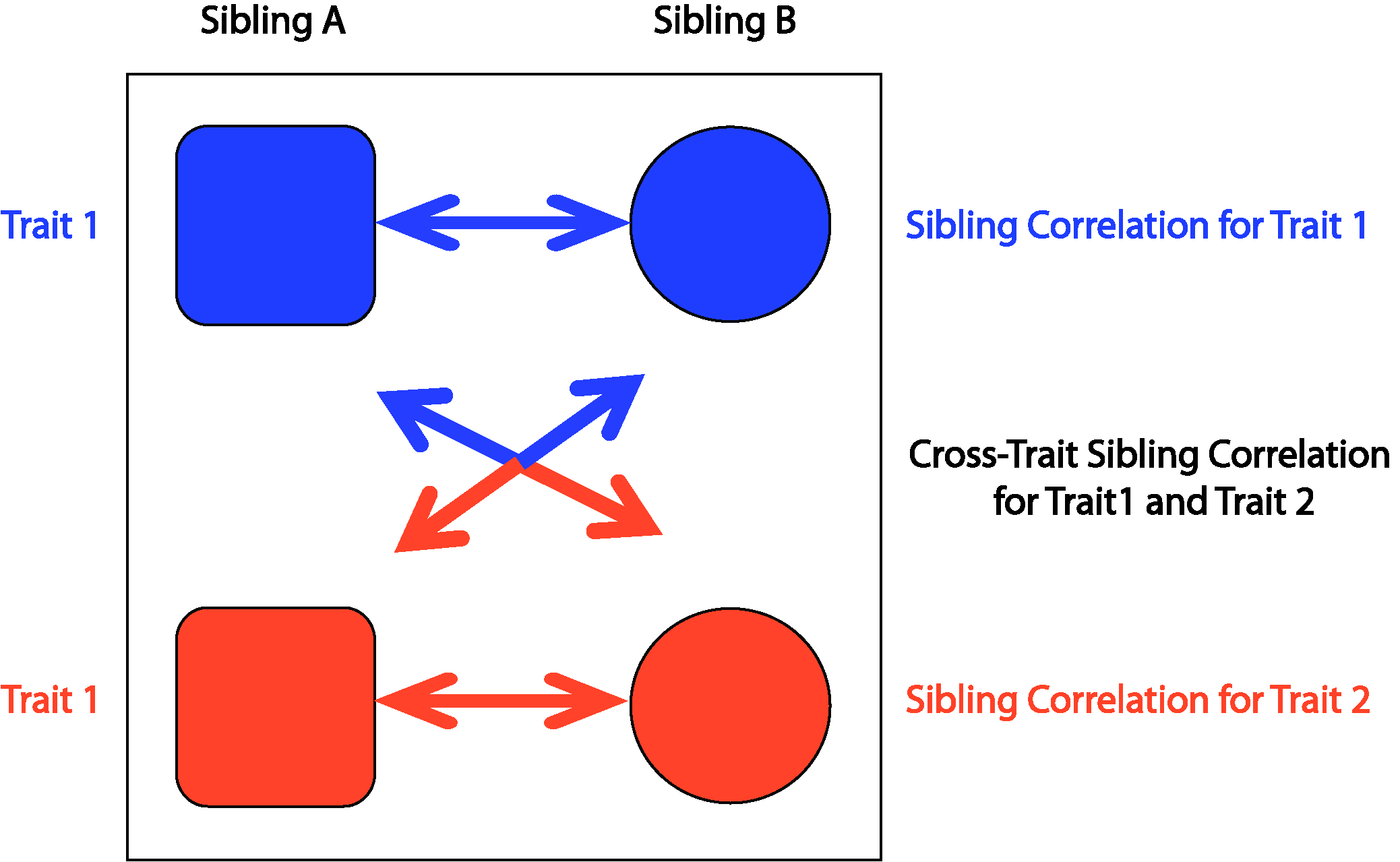

Supplement: Figure S3 — Heritability and co-heritability estimation. The heritability of a trait is derived from the correlation among siblings, as shown in the diagram by the solid blue and orange arrows. Co-heritability of two traits is derived from the cross-trait sibling correlation which is obtained by averaging the correlation of the first trait in sibling A with the second trait in sibling B and the correlation of the second trait in sibling A with the first trait in sibling B shown by the crossed blue and orange arrows. (TIF) [file pone.0043728.s003.tif]

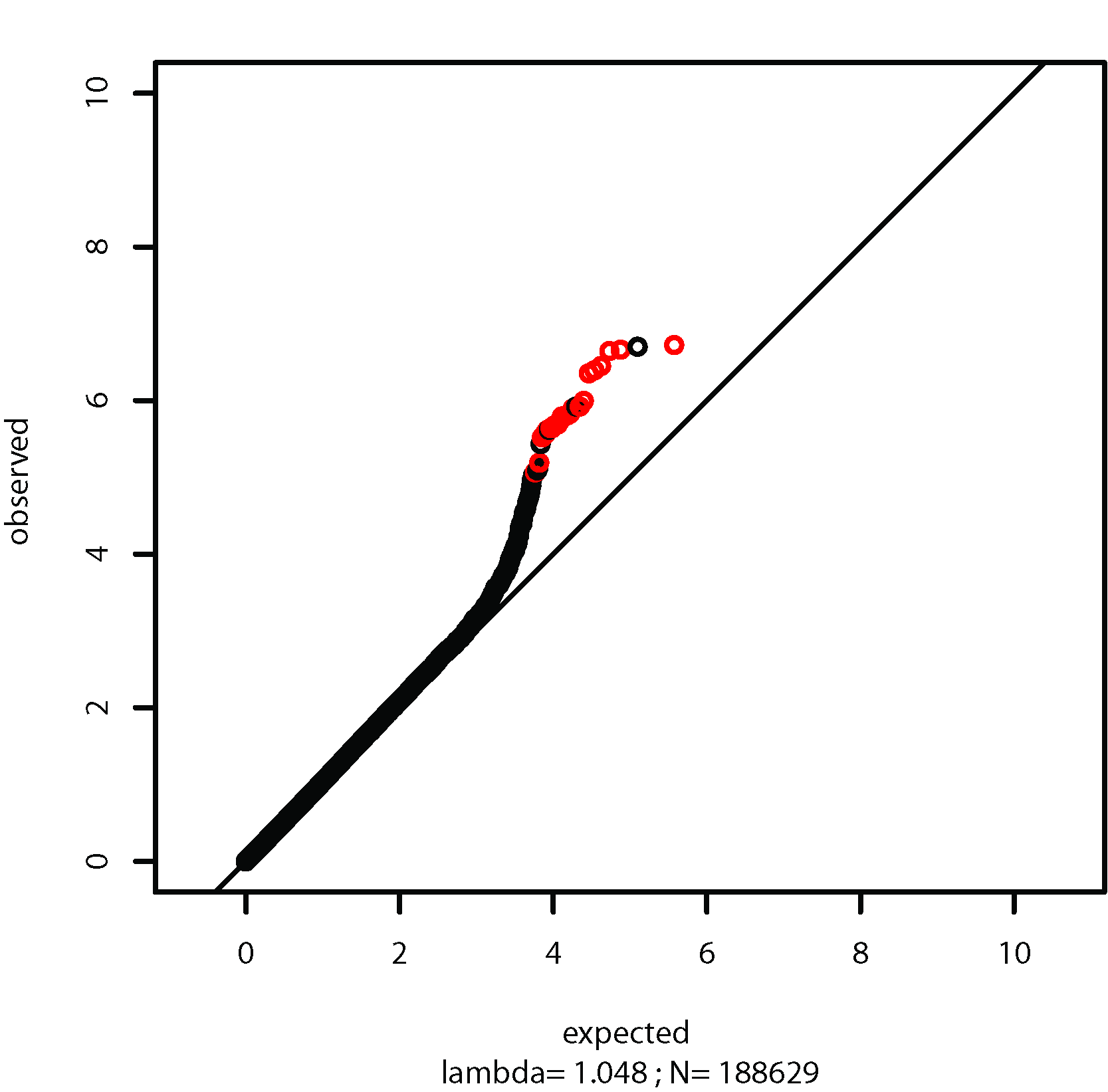

Supplement: Figure S4 — Quantile-quantile (Q-Q) plot of observed (y-axis) vs. expected (x-axis) P-values from genome-wide association tests for the bivariate outcome of cortical cataract and temporal horn volume. Black dots represent all genotyped SNPs and red dots denote the imputed SNPs from CTNND2 with P<10−5. The number of SNPs (188,629) includes genotyped SNPs (186,192) and imputed SNPs (2,437) from selected gene regions. (TIF) [file pone.0043728.s004.tif]

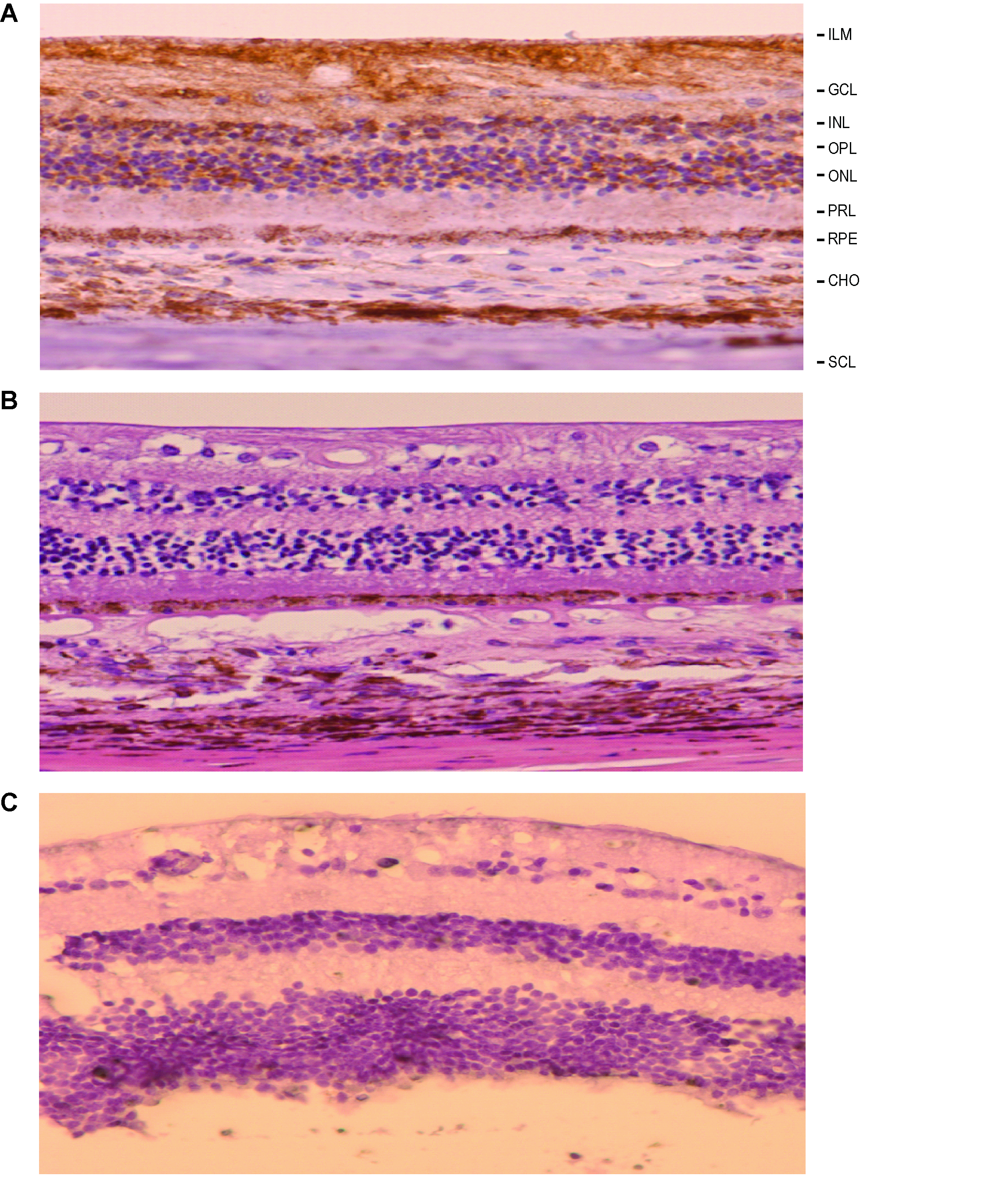

Supplement: Figure S5 — δ-catenin in the human retina. A. δ-catenin immunostianing in a retina from a 70-year-old female with AD. B. same retina in panel A stained with hematoxylin and eosin. C. δ-catenin immunostianing in a retina from a 50-year-old male control. Abbreviations: VIT, vitreous body; ILM, inner limiting membrane; GCL, granule cell layer; INL, inner nuclear layer; OPL, outer plexiform layer; ONL, outer nuclear layer; PRL, photoreceptor layer; RPE, retinal pigment epithelium; CHO, choroid; SCL, sclera. (TIF) [file pone.0043728.s005.tif]
