# Supplementary material for: δ-Catenin Is Genetically and Biologically Associated with Cortical Cataract and Future Alzheimer-Related Structural and Functional Brain Changes
Source: PLoS One. 2012 Sep 11;7(9):e43728. doi: 10.1371/journal.pone.0043728 (PMC3439481; doi:10.1371/journal.pone.0043728)
Supplement: Table S1 — Sample characteristics. (DOCX) [file pone.0043728.s006.docx]

**Table S1.** Sample characteristics

| **Characteristics** | | | **Offspring Cohort** | **Original Cohort** |
| --- | --- | --- | --- | --- |
| **Subjects with MRI Data** | | |  |  |
|  | Number of Subjects at First MRI Exam | | 2130 | 249 |
|  | Number of Subjects at Second MRI Exam | | 1393 | 151 |
|  | Number of Incident AD Cases | | 16 | 39 |
|  | Number of Dementia at First MRI Exam | | 0 | 0 |
|  | Number of Stroke at First MRI Exam | | 11 | 8 |
|  | % Female | | 53.2 | 65.5 |
|  | Age at Enrollment^*^ | | 34.07 (9.56) | 34.37 (3.77) |
|  | Age at DNA Collection^*^ | | 59.56 (9.51) | 82.63 (3.50) |
|  | Age at First MRI Exam^*^ | | 61.25 (9.34) | 84.05 (5.28) |
|  | Interval Between First and Second MRI Exams^*^ | | 6.15 (1.11) | 2.63 (1.08) |
|  | Baseline Frontal Lobar Volume (FBV)^*^ | | 457.67 (60.10) | 398.99 (51.60) |
|  | Baseline Occipital Brain Volume (OBV)^*^ | | 137.81 (40.46) | 127.32 (35.28) |
|  | Baseline Parietal Brain Volume (PBV)^*^ | | 272.60 (48.42) | 240.34 (39.82) |
|  | Baseline Temporal Brain Volume (TBV)^*^ | | 132.15 (17.44) | 112.26 (15.88) |
|  | Baseline Hippocampal Volume (HPV)^*^ | | 4.21 (0.71) | 3.39 (0.68) |
|  | Baseline Lateral Ventricular Volume (LVV)^*^ | | 23.28 (15.63) | 46.32 (22.04) |
|  | Baseline Temporal Horn Volume (THV)^*^ | | 0.68 (0.63) | 2.14 (1.53) |
|  | Baseline White Matter Hyperintensity Volume (WMHV)^*^ | | 1.25 (2.78) | 7.26 (8.80) |
|  | Annual Change for FBV^*^ | | -2.82 (4.94) | -1.77 (3.97) |
|  | Annual Change for OBV^*^ | | 1.13 (6.11) | 2.13 (4.66) |
|  | Annual Change for PBV^*^ | | -1.91 (7.34) | -2.21 (5.61) |
|  | Annual Change for TBV^*^ | | -1.91 (1.48) | -1.04 (1.18) |
|  | Annual Change for HBV^*^ | | 0.07 (0.10) | -0.04 (0.08) |
|  | Annual Change for LVV^*^ | | 0.99 (0.93) | 0.80 (0.93) |
|  | Annual Change for THV^*^ | | 0.03 (0.08) | 0.01 (0.09) |
|  | Annual Change for WMHV^*^ | | 0.21 (0.41) | 0.42 (0.52) |
| **Subjects With Eye Data**  Number of Subjects  Number of Incident AD Cases  Cortical Cataract (CC) Score^*^  Posterior Subcapsular Score^*^  Nuclear Cataract (NC) Score^*^ | | | 2123  18  0.23 (0.69)  1.11 (0.34)  0.16 (0.80) | 739  121  1.16 (1.30)  1.75 (0.79)  (0.92 (2.10) |
| **Subjects with MRI and Eye Data** | | |  |  |
|  | | Number of Subjects | 1249 | NA |
|  | | Number of Incident AD Cases | 7 | NA |
|  | | Number of Dementia at First MRI Exam | 0 | NA |
|  | | Number of Stroke at First MRI Exam | 9 | NA |
|  | | Temporal Horn Volume at First Exam^*^ | 0.66 (0.57) | NA |
|  | | % Female | 53 | NA |
|  | | Age at Enrollment^*^ | 42.96 (7.02) | NA |
|  | | Age at DNA Collection^*^ | 68.14 (6.92) | NA |
|  | | Age at Eye Exam^*^ | 51.31 (8.97) | NA |
|  | | Interval Between Eye and Baseline MRI Exams^*^ | 9.27 (0.38) | NA |
|  | | CC Score^*^ | 0.13 (0.50) | NA |
|  | | PSC Score^*^ | 0.16 (0.85) | NA |
|  | | NC Score^*^ | 1.05 (0.24) | NA |

^*^ Mean (Standard Deviation)

# unit of volume in cm^3^

NA = not applicable
