# Supplementary material for: δ-Catenin Is Genetically and Biologically Associated with Cortical Cataract and Future Alzheimer-Related Structural and Functional Brain Changes
Source: PLoS One. 2012 Sep 11;7(9):e43728. doi: 10.1371/journal.pone.0043728 (PMC3439481; doi:10.1371/journal.pone.0043728)
Supplement: Table S2 — Trait correlations. (DOCX) [file pone.0043728.s007.docx]

**Table S2.** Trait correlations

| **Traits**^*^ | **Cross-Trait Correlation with AD** | | | | | | |  | **Cross-Trait Correlation with CC** | | | | | | |
| --- | --- | --- | --- | --- | --- | --- | --- | --- | --- | --- | --- | --- | --- | --- | --- |
|  | **Within Individuals**^†^ | | |  | **Between Siblings**^‡^ | | |  | **Within Individuals**^†^ | | |  | **Between Siblings**^‡^ | | |
|  | **N** | **COR** | **P** |  | **Sibpairs** | **COR** | **P** |  | **N** | **COR** | **P** |  | **Sibpairs** | **COR** | **P** |
| **Alzheimer Disease** | 5613^ | 1.000 | --- |  | 2779 | 0.199 | < 10^-4^ |  | 1003 | 0.098 | 0.096 |  | 571 | 0.088 | 0.019 |
| **MRI at Baseline:** |  |  |  |  |  |  |  |  |  |  |  |  |  |  |  |
| FBV | 2364 | -0.127 | 1 x 10^-4^ |  | 1377 | -0.089 | 0.042 |  | 1251 | -0.113 | 0.0023 |  | 741 | -0.103 | 0.021 |
| OBV | 2364 | -0.022 | ns |  | 1377 | -0.022 | ns |  | 1251 | -0.024 | ns |  | 741 | -0.043 | Ns |
| PBV | 2364 | -0.098 | 0.002 |  | 1377 | -0.041 | ns |  | 1251 | 0.018 | ns |  | 741 | -0.024 | Ns |
| TBV | 2364 | -0.269 | < 10^-4^ |  | 1377 | -0.145 | 6 x 10^-4^ |  | 1251 | -0.074 | 0.046 |  | 741 | -0.085 | 0.0498 |
| HPV | 2364 | -0.200 | < 10^-4^ |  | 1354 | -0.099 | 0.025 |  | 1236 | -0.103 | 0.004 |  | 774 | -0.026 | Ns |
| LVV | 2364 | 0.233 | < 10^-4^ |  | 1377 | 0.136 | 0.001 |  | 1251 | 0.096 | 0.019 |  | 741 | 0.101 | 0.020 |
| THV | 2364 | 0.350 | < 10^-4^ |  | 1377 | 0.159 | 1 x 10^-4^ |  | 1251 | 0.121 | 0.022 |  | 741 | 0.107 | 0.024 |
| WMHV | 2364 | 0.263 | < 10^-4^ |  | 1377 | 0.114 | 0.006 |  | 1251 | 0.098 | 0.080 |  | 741 | 0.110 | 0.057 |
| **MRI Annual Change:** | |  |  |  |  |  |  |  |  |  |  |  |  |  |  |
| FBV | 1536 | 0.018 | ns |  | 906 | 0.040 | ns |  | 932 | 0.004 | ns |  | 545 | -0.062 | ns |
| OBV | 1536 | -0.017 | ns |  | 906 | -0.006 | ns |  | 932 | 0.043 | ns |  | 545 | 0.012 | ns |
| PBV | 1536 | 0.017 | ns |  | 906 | -0.010 | ns |  | 932 | -0.059 | ns |  | 545 | 0.008 | ns |
| TBV | 1536 | -0.010 | ns |  | 906 | 0.053 | ns |  | 932 | 0.006 | ns |  | 545 | 0.008 | ns |
| HPV | 596 | -0.110 | ns |  | 370 | -0.019 | ns |  | 360 | 0.037 | ns |  | 235 | -0.088 | ns |
| LVV | 1536 | 0.046 | ns |  | 906 | -0.004 | ns |  | 932 | 0.056 | ns |  | 545 | 0.091 | 0.035 |
| THV | 1536 | 0.028 | ns |  | 906 | 0.025 | ns |  | 932 | 0.043 | ns |  | 545 | 0.073 | ns |
| WMHV | 1536 | 0.098 | 0.041 |  | 906 | 0.083 | ns |  | 932 | 0.148 | 7 x 10^-4^ |  | 545 | 0.098 | 0.023 |
| **Cataract:** |  |  |  |  |  |  |  |  |  |  |  |  |  |  |  |
| NC | 2753 | 0.189 | < 10^-4^ |  | 1544 | 0.068 | 0.021 |  | 2132 | 0.213 | < 10^-4^ |  | 1006 | 0.068 | 0.048 |
| CC | 2743 | 0.186 | < 10^-4^ |  | 1544 | 0.121 | < 10^-4^ |  | 2132 | 1.000 | --- |  | 1006 | 0.174 | 2 x 10^-4^ |
| PSC | 2805 | 0.136 | < 10^-4^ |  | 1557 | 0.113 | 1 x 10^-4^ |  | 2132 | 0.222 | < 10^-4^ |  | 1009 | 0.066 | 0.021 |

N= number of subjects; COR = correlation estimate; P = p-value; Sibpairs = number of sibpairs; ns = not significant at P > 0.1; AD = Alzheimer disease; MRI Annual Change = annualized change in value of MRI measure between baseline exam and follow-up exam; FBV = frontal lobar volume; OBV = occipital brain volume; PBV = parietal brain volume; TBV = temporal brain volume; HPV = hippocampal volume; THV = temporal horn volume; LVV = lateral ventricular volume; WMHV = white matter hyperintensity volume; CC = cortical cataract, NC = nuclear cataract, PSC = posterior subscapular cataract.

^*^ All trait values before adjustment and normalization. ^†^ Correlation within an individual. ^ includes 61 AD cases

^‡^ Cross-trait correlation between siblings was calculated by averaging the correlation of trait 1 in sib A with trait 2 in sib B, and the correlation of trait 1 in sib B with trait 2 in sib A
