# Supplementary material for: δ-Catenin Is Genetically and Biologically Associated with Cortical Cataract and Future Alzheimer-Related Structural and Functional Brain Changes
Source: PLoS One. 2012 Sep 11;7(9):e43728. doi: 10.1371/journal.pone.0043728 (PMC3439481; doi:10.1371/journal.pone.0043728)
Supplement: Table S3 — Correlations for cortical cataract with selected MRI traits. (DOCX) [file pone.0043728.s008.docx]

**Table S3. C**orrelations for cortical cataract with selected MRI traits.

| **Trait**^*^ | **Cross-Trait Correlation (P value)^†^** | | | | | | |
| --- | --- | --- | --- | --- | --- | --- | --- |
|  | **CC** | **THV** | **LVV** | **HPV** | **arTHV** | **arLVV** | **arHPV** |
| **CC** | **0.360 (< 10^-4^)** | 0.239 (<10^-4^) | 0.153 (5 x 10^-4^) | -0.094 (0.028) | -0.071 (0.137) | -0.095 (0.054) | -0.081 (0.249) |
| **THV** | 0.318 (<10^-4^) | **0.316 (< 10^-4^)** | 0.208 (1 x 10^-4^) | -0.037 (0.334) | -0.023 (0.580) | 0.004 (0.926) | -0.115 (0.068) |
| **LVV** | 0.127 (1 x 10^-4^) | 0.573 (1 x 10^-4^) | **0.352 (< 10^-4^)** | -0.062 (0.405) | 0.087 (0.031) | 0.142 (6 x 10^-4^) | -0.059 (0.355) |
| **HPV** | -0.090 (0.004) | -0.025 (0.263) | -0.054 (0.016) | **0.340 (< 10^-4^)** | -0.034 (0.388) | 0.001 (0.984) | 0.157 (0.012) |
| **arcTHV** | -0.128 (0.001) | -0.033 (0.238) | 0.240 (<10^-4^) | 0.058 (0.028) | **0.145 (0.012)** | 0.171 (3 x 10^-4^) | 0.038 (0.535) |
| **arcLVV** | -0.148 (2 x 10^-4^) | 0.057 (0.043) | 0.352 (<10^-4^) | 0.060 (0.025) | 0.619 (<10^-4^) | **0.194 (4 x 10^-4^)** | -0.024 (0.714) |
| **arcHPV** | -0.126 (0.024) | -0.192 (<10^-4^) | -0.151 (3 x 10^-4^) | -0.367 (<10^-4^) | -0.046 (0.247) | 0.010 (0.804) | **0.354 (0.001)** |

CC: cortical cataract; NC: nuclear cataract; PSC: posterior subscapular cataract; THV: temporal horn volume; LVV: lateral ventricular volume; arc: annual rate of change.

^*^ All traits were normalized and adjusted for age and sex.

^†^ Within individual cross-trait correlations are presented below the diagonal (clear cells). Sibling cross-trait correlations are shown above the diagonal (shaded cells). Heritability (h^2^) is derived from the sibling correlation (ρ, shown as bolded values on the diagonal), where h^2^ = 2ρ.
