# Supplementary material for: δ-Catenin Is Genetically and Biologically Associated with Cortical Cataract and Future Alzheimer-Related Structural and Functional Brain Changes
Source: PLoS One. 2012 Sep 11;7(9):e43728. doi: 10.1371/journal.pone.0043728 (PMC3439481; doi:10.1371/journal.pone.0043728)
Supplement: Table S4 — Top-ranked GWAS results (P<10−5) with 186,192 genotyped SNPs in bivariate models of cataract and temporal horn volume. (DOCX) [file pone.0043728.s009.docx]

**Table S4.** Top-ranked GWAS results (P < 10^-5^) with 186,192 genotyped SNPs in bivariate models of cataract and temporal horn volume

| SNP | CH | Position (BP) | Gene | Alleles | MA | MAF | HWP | P value for Bivariate Model^*^ | | |
| --- | --- | --- | --- | --- | --- | --- | --- | --- | --- | --- |
|  |  |  |  |  |  |  |  | P_CC-THV_ | P_NC-THV_ | P_PSC-THV_ |
| rs16901246 | 5 | 11,089,821 | *CTNND2* | G/T | T | 0.111 | 0.80 | 3.5 x 10^-6^ | 0.14 | 0.16 |
| rs13184045 | 5 | 11,100,668 | *CTNND2* | C/T | T | 0.105 | 0.79 | 3.7 x 10^-6^ | 0.13 | 0.13 |
| rs17787646 | 5 | 11,105,907 | *CTNND2* | A/G | G | 0.106 | 0.89 | 1.2 x 10^-6^ | 0.16 | 0.12 |

CH: chromosome; BP: base pair; MA: minor allele; MAF: minor allele frequency; HWP: Hardy-Weinberg P value; CC = cortical cataract; NC = nuclear cataract; PSC = posterior subcapsular cataract; THV = baseline temporal horn volume.

^*^ Traits were normalized after adjustment for age and gender.
