# Supplementary material for: δ-Catenin Is Genetically and Biologically Associated with Cortical Cataract and Future Alzheimer-Related Structural and Functional Brain Changes
Source: PLoS One. 2012 Sep 11;7(9):e43728. doi: 10.1371/journal.pone.0043728 (PMC3439481; doi:10.1371/journal.pone.0043728)
Supplement: Table S5 — Top-ranked association results (P<10−5) for CTNND2 SNPs with cortical cataract (CC), temporal horn volume (THV), and the bivariate outcome CC-THV. (DOCX) [file pone.0043728.s010.docx]

**Table S5.** Top-ranked association results (P < 10^-5^) for CTNND2 SNPs with cortical cataract (CC), temporal horn volume (THV), and the bivariate outcome CC-THV.

RA: reference allele; RAF: reference allele frequency; RSQ: squared correlation of imputed and actual genotypes; β: effect size

SNP map positions and gene annotations obtained from HG19 release of the UCSC genome browser (<http://genome.ucsc.edu>) and dbSNP build 130 (<http://www.ncbi.nlm.nih.gov/snp>). Genotyped SNPs are underlined and italicized. Genome wide significant results are shown in bold text.
